# Changes in Resilience Following Engagement With a Virtual Mental Health System: Real-world Observational Study

**DOI:** 10.2196/37169

**Published:** 2022-07-29

**Authors:** Grant Graziani, Brandon S Aylward, Sarah Kunkle, Emily Shih

**Affiliations:** 1 Ginger San Francisco, CA United States

**Keywords:** behavioral coaching, psychological resilience, mental health, telehealth

## Abstract

**Background:**

Digital health services can serve as scalable solutions to address the growing demand for mental health care. However, more research is needed to better understand the association between engagement with care and improvements in subclinical outcomes.

**Objective:**

This study aims to fill this research gap by examining the relationship between members’ engagement with the Ginger platform and changes in their psychological resilience.

**Methods:**

We conducted a retrospective observational study of 3272 members who accessed Ginger, an on-demand mental health service, between January 2021 and November 2021. Each member completed the 10-item Connor-Davidson Resilience Scale questionnaire, a measure of psychological resilience, at baseline and again during a 6- to 16-week follow-up window. Depression and anxiety symptoms (9-item Patient Health Questionnaire and 7-item Generalized Anxiety Disorder) were also measured. Linear regression was used to identify the association between engagement with Ginger’s multiple care modalities and changes in resilience. Moderator analysis was conducted to test whether clinical depression or anxiety at baseline moderated the relationship between engagement level and changes in resilience.

**Results:**

Of the 3272 members, 2683 (82%) reported low resilience at baseline. The mean change in resilience was 0.77 (SD 5.50) points. Linear regression models showed that age and census region did not predict changes in resilience; however, male members showed larger improvements (coefficient=0.58; *P*=.04). Baseline mental health outcomes, including resilience and depression and anxiety symptoms, were strong predictors of changes in resilience. Every point decrease in baseline resilience is associated with a 0.28-point increase in change in resilience (*P*<.001), and members with no or mild depression and anxiety at baseline saw changes in resilience that were 1.44 points (*P*<.001) larger than their clinical counterparts. Engagement with the Ginger system predicted changes in resilience. Members who engaged with Ginger coaching, clinical services, or both improved their resilience by 1.82, 1.55, and 1.40 points, respectively (*P*<.001), more than those who only engaged with Ginger content. Screening negative for moderate to severe depression and anxiety at baseline was associated with larger improvements in resilience (coefficient=1.30; *P*<.001); however, subclinical status was not shown to be a moderator for the association between level of engagement and changes in resilience.

**Conclusions:**

Engagement with Ginger services was associated with improvements in resilience. Members who engaged in coaching or clinical care had significantly larger improvements compared with those who only engaged in self-guided content, regardless of whether a member screened positive for clinical depression or anxiety at baseline.

## Introduction

### Background

Resilience is a multidimensional construct that may be viewed as one’s ability to cope with stress or represent personal qualities that enable one to thrive in the face of adversity [1-3]. Resilience has been studied in a variety of disciplines, including psychology, psychiatry, sociology, genetics, and neuroscience among others [4,5]. The definition of resilience has evolved along with increased scientific knowledge in these disciplines. As such, researchers have argued that resilience is “the process of multiple biological, psychological, social, and ecological systems interacting in ways that help individuals to regain, sustain, or improve their mental wellbeing when challenged by one or more risk factors” [6]. Furthermore, research has shown that resilience can vary with factors such as time, context, gender, and cultural origin [7]. From a strength-based perspective, resilience can contribute to positive functioning and help prevent negative emotions, thoughts, and behaviors [8]. Studies have demonstrated that resilience is a mitigating factor for heightened stress and adverse mental health outcomes in life circumstances.[9]. Specifically, individuals with low or normal levels of resilience have been found to be more likely to experience mental distress than those with high resilience [10,11].

Although many researchers’ conceptualization of resilience is somewhat related to the notions mentioned earlier, namely, individuals’ ability to cope with stress and adverse circumstances, there is no consensus on the operational definition of resilience according to a recent meta-analysis [12]. Given this, studies such as this one must be explicit about the conceptualization of resilience used as well as the precise measurement strategy. For the purpose of this study, psychological resilience is defined as how well one is able to adapt to change and *bounce back* after stressful events, tragedy, or trauma. This framework is consistent with the 10-item Connor-Davidson Resilience Scale (CD-RISC-10), which is the survey instrument used to measure resilience in this study. The 10 topics included in the CD-RISC-10 were confidence, determination, flexibility, focus, grit, perseverance, personal growth, positivity, self-reliance, and weathering emotions. This measure is explained in more detail in the *Data* section.

In light of the current pandemic, there has been increased focus on employee mental health and recognition of the importance of resilience in daily stress management and overall well-being [13]. For many, the COVID-19 pandemic fundamentally changed how and where one works as well as the daily demands of their jobs, and many reported increased loneliness, anxiety, depression, and suicidal ideation [14-16]. Recent studies have found that individuals with lower resilience scores are more likely to experience mental distress and express greater difficulty coping with the emotional challenges of the COVID-19 pandemic [10,16]. Similarly, a population-based study in China at the peak of the pandemic found that psychological resilience was significantly negatively correlated with depression, anxiety, and somatization symptom scores [17]. In addition, a study among public workers found that resilience mediated the effect of depression in public workers and their stress and anxiety levels during the pandemic [18].

Behavioral health coaching draws from several theoretical approaches that can effectively impact resilience or well-being. Coaching interventions address a variety of day-to-day challenges by identifying and working toward concrete and actionable goals. Resilience is seen as a proactive capability that supports the attainment of such goals and enhances overall mental health [19]. During the pandemic and postpandemic return to the office, many organizations have allocated increased resources toward the mental health and well-being of their employees, including resilience training and coaching-based interventions. However, a review of resilience intervention studies found that most in-person trainings were of short duration and had limited follow-up periods [13].

Previous studies have indicated a positive relationship between resilience and well-being, with higher resilience in the workplace setting associated with better mental health, reduced stress, and greater well-being [13]. Furthermore, the authors found that individuals who participated more often in the web-based resilience training program achieved the greatest improvements [13]. A recent multilevel meta-analysis found that resilience-promoting interventions yielded a small but significant overall effect on resilience [12]. One key finding was that ambiguity in conceptualizing and operationalizing resilience, in turn, leads to variability both between and within treatment effect sizes [12].

In addition to resilience-focused interventions, studies have found that general coaching interventions have also demonstrated that coaching can support resilience, even in the absence of it being the focus or aim of services. For example, a randomized controlled study of executives in a public health agency found that individual coaching sessions enhanced goal attainment, increased resilience and workplace well-being, and decreased depression and stress compared with controls [20]. Similarly, Lee et al [21] found that a health coaching program is an effective strategy for improving resiliency in youth.

Ginger offers various types of care designed to provide mental health support, including self-guided content, text-based behavioral health coaching, teletherapy, and telepsychiatry. Theoretically, each modality of care has a different effect on resilience. This hypothesis was tested in this study. Furthermore, given the clinical focus of therapy and psychiatry, these modalities may have different impacts on resilience depending on whether a member presents with clinical symptoms. It could be that these modalities are more effective at impacting resilience for these members if interventions designed to impact clinical symptoms are more impactful on resilience. Alternatively, if addressing clinical symptoms is the focus of care before addressing subclinical outcomes such as resilience, we could expect to see that therapy and psychiatry have a smaller impact on resilience (but perhaps have an equal or larger impact on a time horizon beyond the scope of this study). Given the unique Ginger context that offers multiple care modalities, testing whether clinical symptoms moderate the impact of engagement on resilience is possible. We are not aware of any existing studies that explicitly test this moderator hypothesis.

Overall, literature supports the relationship between resilience and other mental health and well-being outcomes and the fact that interventions, including coaching, can bolster resilience. Given that most of these studies have been conducted in controlled research settings, it is important to supplement this knowledge to better understand what is happening in real-world settings, particularly when a global pandemic introduces unique challenges to resilience.

### Study Objectives

The purpose of this study was to examine changes in resilience among members seeking on-demand mental health treatment. We explicitly tested the following three hypotheses:

1. Change in resilience is associated with member characteristics at baseline, including demographic characteristics and baseline mental health outcomes (baseline resilience, depression symptoms, and anxiety symptoms).
2. Engagement with Ginger care is associated with larger improvements in resilience.
3. Baseline depression and anxiety symptoms moderate the association between engagement and changes in resilience.

Consistent with previous literature [10,11,13,17], we hypothesize that resilience will increase over the follow-up time points of treatment, and those with higher anxiety or depression symptom scores at intake will evidence smaller improvements or worsening in resilience over time.

### Study Contributions

This study contributes to the literature on resilience in several ways. First, we present the results of one of the largest longitudinal studies of resilience. Our sample includes 3272 individuals. Second, to our knowledge, this is the first study to specifically examine resilience in the context of a digital mental health system that offers self-guided content, text-based behavioral health coaching, telepsychotherapy, and telepsychiatry. Third, by leveraging our rich data on Ginger members, we tested specific hypotheses that relate resilience to clinical depression and anxiety symptoms. In particular, we were able to test for the first time whether depression and anxiety symptoms moderate the impact of coaching and clinical interventions on resilience.

## Methods

### Overview

This was a retrospective observational study of Ginger members: individuals who joined Ginger, an on-demand mental health system. Data were collected between January 1, 2021, and November 13, 2021, from Ginger members residing in the United States. As part of its measurement-based care system, Ginger used the CD-RISC-10 as an indicator of resilience at intake as well as to track treatment progress beyond anxiety and depression symptom scores. By leveraging a retrospective design, this study contributes to the growing literature using real-world evidence. Although such studies often lack clear causal inference, they offer increased feasibility, larger samples, and robust external validity.

### Participants

Study participants had access to the Ginger system as part of their employee or health plan benefits. Internal clinical protocols include the following exclusionary criteria, where self-directed telehealth is not likely appropriate and more specialized and urgent psychiatric services are required: (1) active suicidal ideation; (2) active high-risk self-harm behavior; (3) 2 or more hospitalizations within the past 6 months or 1 hospitalization in the past month for psychiatric reasons; (4) certain symptoms of psychosis that are poorly managed (eg, member is not medication compliant or symptoms are unresponsive to treatment) and are likely incompatible with telehealth; (5) a primary diagnosis of a substance use disorder or moderate to severe substance abuse issues, owing to the high complexity, severity, and risk frequently associated with such members, as well as the need for specialized care; (6) active eating disorders with symptoms considered high-risk; (7) ongoing grave disability, including certain patients who are bipolar with active mania or hypomania or mixed episodes who are unmedicated or have poor compliance with a medication regimen over time; and (8) two or more medical hospitalizations in the last month, owing to the high likelihood that the individual has a poorly controlled medical condition that requires close monitoring. For this study, we included Ginger users aged ≥18 years who downloaded the app during the data collection period.

### Data

#### The Ginger System

Ginger provides virtual on-demand mental health services, primarily through employee or health plan benefits. Using a mobile app platform, Ginger members can access text-based behavioral health coaching, teletherapy, and telepsychiatry, as well as self-guided content and assessments. Individuals who are eligible for Ginger can download the mobile app, complete an onboarding process, and begin texting with a behavioral health coach within minutes. Members who are interested in or have been determined to be in need of a higher level of care can meet with a therapist or psychiatrist via video. All participants had access to self-care activities via mobile apps. Additional details regarding the Ginger system can be found in prior publications evaluating depression and anxiety outcomes as measured by the 9-item Patient Health Questionnaire (PHQ-9) and 7-item Generalized Anxiety Disorder (GAD-7) surveys [22,23].

#### Data Collection

**Figure 1:**
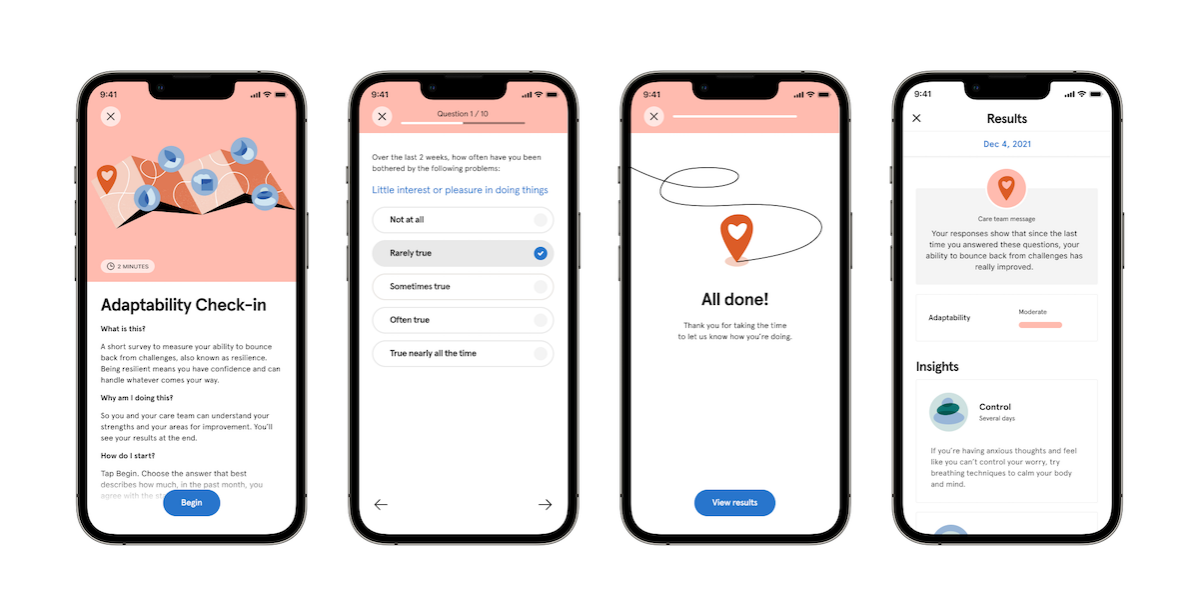
The 10-item Connor-Davidson Resilience Scale survey in the Ginger mobile app.

Ginger uses various assessments including the PHQ-9 and GAD-7 surveys as part of its measurement-based care system. Since December 2020, Ginger has used the CD-RISC-10 survey (referred to as an adaptability check-in within the app) to track progress beyond depression and anxiety symptom scores. This is particularly relevant to understand the needs of *subclinical* members (ie, members who do not exhibit clinically significant levels of depression or anxiety at intake). The CD-RISC-10 was selected because of its focus on behavioral health coaching to build resilience and its strength-based focus. A total of 7 CD-RISC-10 surveys were sent to members 1 week after enrollment, and follow-up surveys were sent to members every 30 days. Importantly, members who signed up but did not engage with the app past the 1-week mark did not complete the baseline survey. In this way, members with a low likelihood of meaningful engagement (a proxy for behavioral health needs) were excluded from the sample. A visual depiction of how the CD-RISC-10 survey appears to the members is shown in Figure 1.

### Measures

#### CD-RISC-10 Assessment Tool

A common assessment tool for resilience is the CD-RISC-10. As mentioned earlier, Ginger uses the CD-RISC-10 as a proxy measure of an individual’s psychological resilience level. The original researchers initially developed a 25-item scale to measure resilience or how well one is able to adapt to change and *bounce back* after stressful events, tragedy, or trauma. A total of 2 brief versions, the 10-item (CD-RISC-10) [24] and the 2-item (CD-RISC-2) [25], were subsequently developed by other research teams. The CD-RISC-10 contains 10 of the original 25 items from the CD-RISC scale and has demonstrated robust validity, reliability, and practicality [24]. The 10 topics included in the CD-RISC-10 were confidence, determination, flexibility, focus, grit, perseverance, personal growth, positivity, self-reliance, and weathering emotions. For each of the 10 items, respondents were asked to rate items on a 5-point scale: not true at all (0), rarely true (1), sometimes true (2), often true (3), and true nearly all the time (4). A respondent’s total score ranges from 0 to 40, with higher scores indicating greater resilience. Results from the US population indicate that the quartiles for this measure are as follows: Q1: 0 to 29, Q2: 30 to 32, Q3: 33 to 36 and Q4: 37 to 40 [22].

#### PHQ-9 Assessments

The PHQ-9 is a 9-item self-report questionnaire that assesses the frequency and severity of depression symptomatology within the previous 2 weeks. Each of the 9 items is based on the Diagnostic and Statistical Manual of Mental Disorders, 4th edition criteria for major depressive disorder and is scored on a 0 (not at all) to 3 (nearly every day) scale. Items include *Little interest or pleasure in doing things* and *Feeling down, depressed, or hopeless.* Total scores range from 0 to 27, with higher scores indicating more depressive symptoms. A score of 10 is used as the clinical threshold [26].

#### GAD-7 Assessments

The GAD-7 is a valid, brief self-report tool used to assess the frequency and severity of anxious thoughts and behaviors over the past 2 weeks. Each of the 7 items is based on the Diagnostic and Statistical Manual of Mental Disorders, 4th edition diagnostic criteria for generalized anxiety disorder and is scored on a 0 (not at all) to 3 (nearly every day) scale, with total scores ranging from 0 to 21. Items include *Feeling nervous, anxious, or on edge* and *Not being able to stop or control worrying*. Consistent with existing literature [27], a score of 10 was used as the clinical threshold for this study.

#### Levels of Engagement

Coaching sessions were operationalized as the number of unique days on which members and coaches each exchanged at least five text messages, the minimum we believe is needed to capture a productive conversation between members and their coaches. Clinical sessions were operationalized as the number of video sessions completed with a clinician.

For this study, 5 different levels of engagement were considered based on members’ engagement with self-guided content, text-based coaching, and teletherapy sessions. Specifically, members who engaged only with self-guided content and did not complete any coaching or clinical sessions were categorized as the *self-guided* group. *Low engagement* was defined as members who completed >4 coaching or >4 teletherapy sessions. Members who completed ≥4 text-based coaching sessions and no clinical sessions were categorized as the *coaching only* group. Members who completed 4 or more clinical teletherapy sessions (with a therapist) and at most one coaching session were categorized as the *clinical only* group. We allow for *clinical only* members to have completed at most one coaching session, given that many members’ point of entry to Ginger is a coaching session, after which they could be escalated to therapy and not continue coaching. Finally, members who completed 4 or more coaching sessions coupled with at least one clinical session or members who completed 4 or more teletherapy sessions coupled with more than 1 coaching session were categorized as the *hybrid care* group. The descriptions and rationales for the creation of these groups are described in Table 1.

**Table 1:** Engagement levels.

Engagement level	Definition	Rationale
Self-guided	Engagement only with self-guided content; 0 coaching and 0 clinical sessions	These are members who have engaged with the app’s self-guided content but have not interacted with any coaching or clinical care. This group serves as the primary reference group in the linear regression models.
Low engagement	Between 0 and 3 total sessions comprising coaching or clinical care	Internal analyses suggest that 4 sessions are an inflection point for meaningful symptom reduction. These are members who have not reached this threshold.
Coaching only	≥4 coaching sessions and 0 clinical sessions	These are members who have completed at least the internally established threshold of 4 sessions but exclusively with coaching care.
Clinical only	≥4 clinical sessions and ≤1 coaching session	These are members who have completed at least the internally established threshold of 4 sessions but exclusively with clinical care.
Hybrid care	>1 coaching session and ≥4 clinical sessions or ≥1 clinical session and ≥4 coaching sessions	These are members who have completed more than the internally established threshold of 4 sessions using a combination of coaching and clinical care.

#### Baseline Characteristics

For each member, the following data were either collected at baseline or were fixed characteristics of members: age group, gender, geographic region, PHQ-9 score, and GAD-7 score. The demographic and location data were not self-reported. Instead, they were reported by a member’s parent organization, which is either their employer or health insurance plan. Baseline PHQ-9 and GAD-7 data were collected using the Ginger system. The baseline PHQ-9 and GAD-7 scores were selected within 1 week before and after a member’s baseline CD-RISC-10 score was collected, and the first PHQ-9 and GAD-7 scores were chosen. Members without baseline PHQ-9 and GAD-7 scores were excluded from analysis.

For many of our participants, the baseline characteristics were missing. The data were missing owing to 1 of 2 reasons. First, a member’s parent organization may not share members’ demographic information. Thus, missing demographic data are a signal of a member’s parent organization and not necessarily a signal of information specific to a given member. For example, of the 197 parent organizations represented in this study, 118 (59.9%) reported all their members’ gender information, 76 (38.6%) reported no gender information, and the remaining 3 (1.5%) organizations reported gender information for some but not all of their members.

### Analyses

#### Sample

The sample for this study included Ginger members residing in the United States who completed a baseline survey between January 1, 2021, and November 13, 2021. Members were excluded from the analysis if they satisfied any of the following criteria:

1. Engagement criterion: Members completed more than 1 coaching session or any number of clinical appointments before their baseline resilience scores. The members’ baseline survey was sent after their first coaching session.
2. Follow-up criterion: A member did not have a follow-up resilience score between 6 and 16 weeks from baseline.
3. PHQ-9 and GAD-7 criteria: Members without valid PHQ-9 and GAD-7 scores within a week of their baseline resilience score.

**Figure 2:**
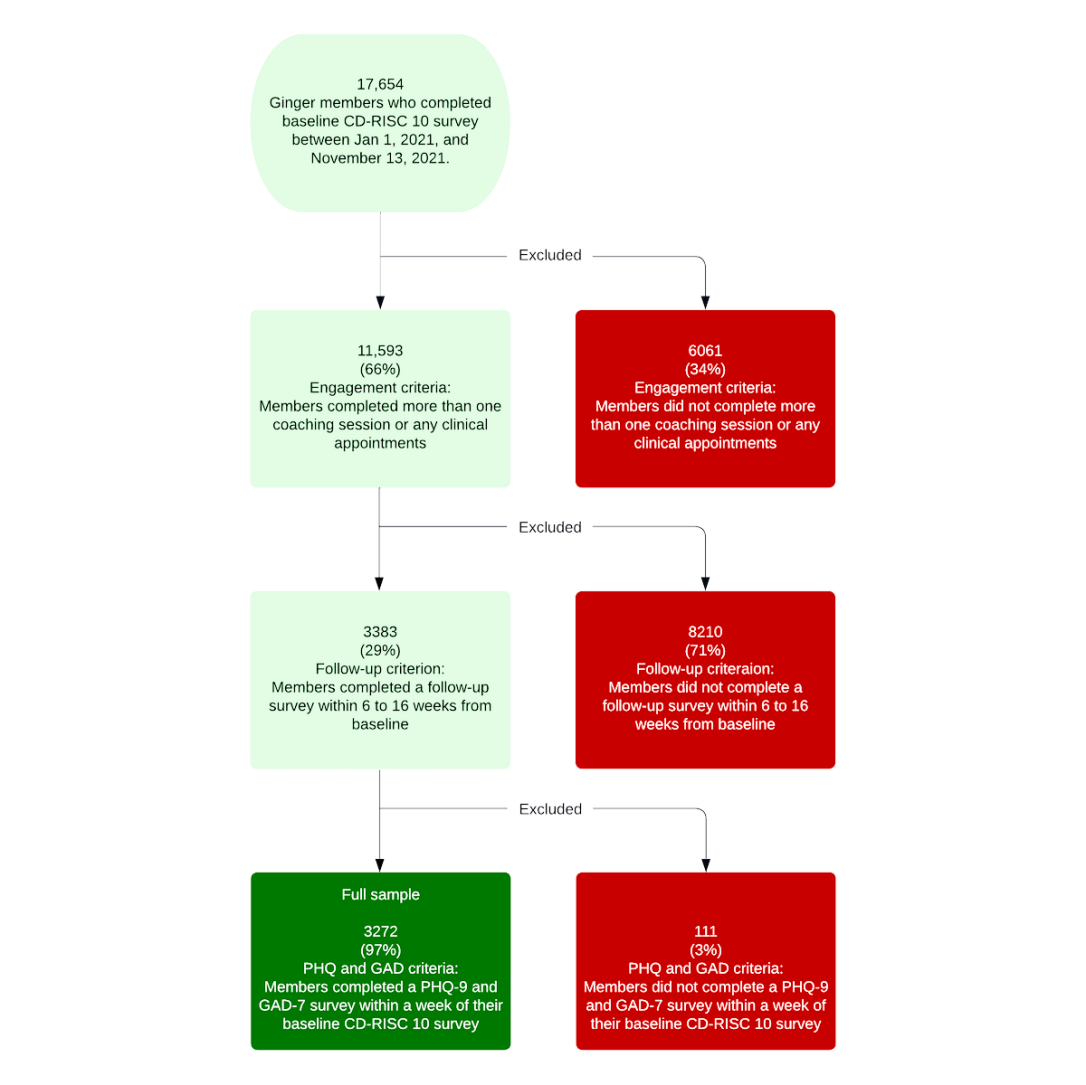
Sample flowchart. CD-RISC-10: 10-item Connor-Davidson Resilience Scale; GAD-7: 7-item Generalized Anxiety Disorder; PHQ-9: 9-item Patient Health Questionnaire.

There were 17,654 members in the baseline resilience survey, of whom 6061 (34.33%) were excluded for meeting the engagement exclusion criteria. Of the remaining 11,593 participants, 3383 (29.18%) completed a follow-up survey between 6 and 16 weeks from their baseline survey. Of these, 3.28% (111/3383) did not have a valid PHQ-9 or GAD-7 score and were thus excluded. The resulting 3272 members comprised the full sample for this study and were used for our descriptive analysis. Of these 3272 members, 2674 (81.72%) had a low baseline score (CD-RISC-10 score<30) and comprised the low-resilience subsample for the study [24]. Given that the Ginger intervention is intended to improve resilience among members with low resilience at baseline, this subsample was used to analyze the association between engagement level and changes in resilience. Figure 2 outlines the sample construction process.

#### Summary Statistics and Subgroup Analysis

Our descriptive analysis summarized the changes in resilience scores by presenting the mean, median, and SD of these change scores. To analyze differences across subgroups of members, we used a 2-tailed Welch 2-tailed *t* test for differences in mean changes across groups with unequal variances when a category had 2 groups (eg, gender). For categories with more than 2 groups (eg, census regions), we used an *F* test as part of an ANOVA to test for significant differences in mean changes across the groups. Furthermore, to understand whether members with missing data had significantly different outcomes than those without missing data, we performed 2-tailed Welch *t* tests to compare mean changes across the missing and nonmissing groups.

The subgroups of focus in this study were based on baseline resilience, depression, and anxiety symptom scores. In particular, members are grouped by their presence at baseline with low resilience (CD-RISC-10 score<30) and moderate to severe depression or anxiety (ie, PHQ-9 or GAD-7 score≥10) [26-28].

#### Descriptive Multivariate Regressions

To understand the association between the demographic and baseline survey responses and changes in resilience, we estimated a multivariate linear ordinary least squares (OLS) regression. The dependent variable for this model was the change in resilience scores. The following categorical independent variables were included in the model: gender, age group, census region, and interacted indicators for whether a member’s baseline PHQ-9 or GAD-7 score was ≥10. For depression and anxiety at baseline, interacted indicators were included in the regression model to account for possible differences based on combinations of depression and anxiety clinical status. Given that missing demographic data are highly dependent on whether a member reports such data for any of their members, indicators for a member’s parent organization were included as independent variables. In addition, given the relatively wide follow-up period (6-16 weeks), indicator variables for the number of weeks between a member’s baseline and follow-up scores were included to account for secular time trends. The coefficients of the indicators for parent organization and weeks between scores have not been reported. For each of these independent variables, a category of members with missing data was included. Homoscedasticity was not assumed, and robust SEs were computed.

#### Moderator Analysis

To understand the association between engagement with Ginger coaching and changes in resilience scores, we leveraged a moderator model with baseline depression and anxiety clinical status as the moderator and the level of engagement category (eg, self-guided, coaching only, and clinical only) as the independent variable. For our moderator categorization, members with either moderate to severe depression or anxiety at baseline were included in the clinical group (ie, PHQ-9 or GAD-7 score≥10), whereas all other members were included in the subclinical group. Clinical status at baseline was the hypothesized moderator of the association between the level of engagement and changes in resilience. We present the mean changes in resilience according to the clinical status for each engagement level. This analysis was restricted to members with low resilience at baseline (ie, CD-RISC-10 score<30).

To formally test whether clinical status at baseline was a moderator for engagement, we used a multivariate OLS regression model that included an indicator for engagement level interacting with an indicator of clinical status at baseline.

### Ethics Approval

This study represents a secondary analysis of pre-existing deidentified data. The study team did not have access to participants or information to identify participants and did not intend to recontact participants. This study protocol was reviewed by Advarra institutional review board and determined to be exempt from institutional review board oversight, as deidentified secondary data analysis is generally not regarded as human subject research.

## Results

### Summary Statistics and Subgroup Analysis

**Figure 3:**
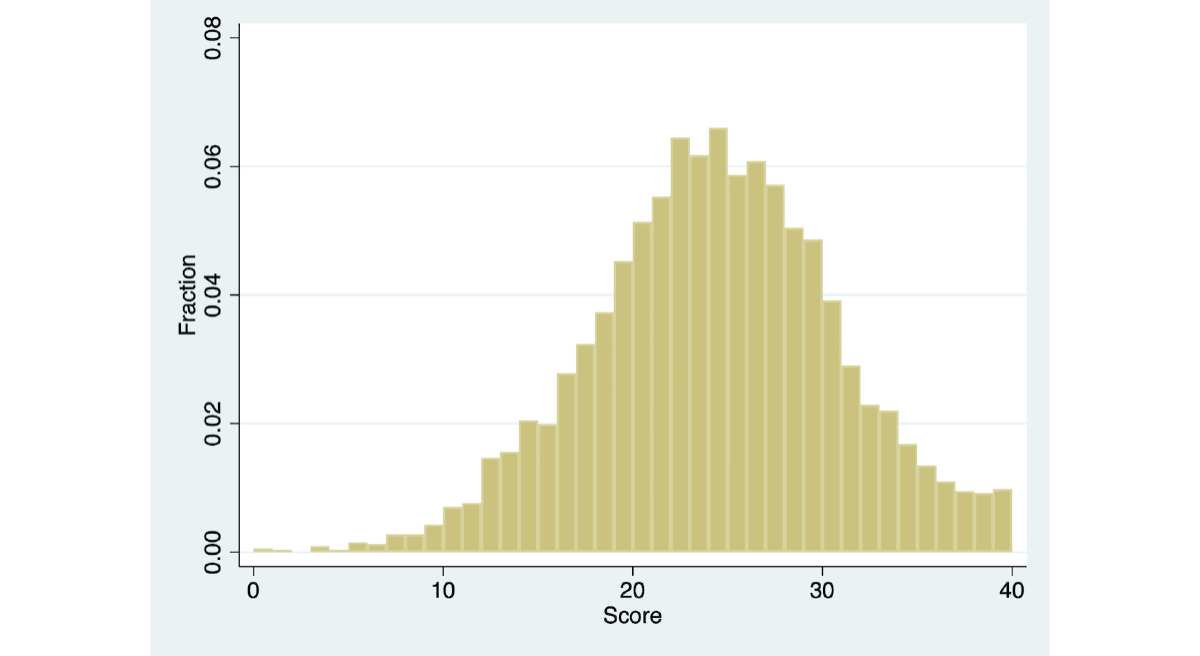
Distribution of baseline resilience (full sample). N=3272; mean=23.83; SD=6.47; median=24.

**Figure 4:**
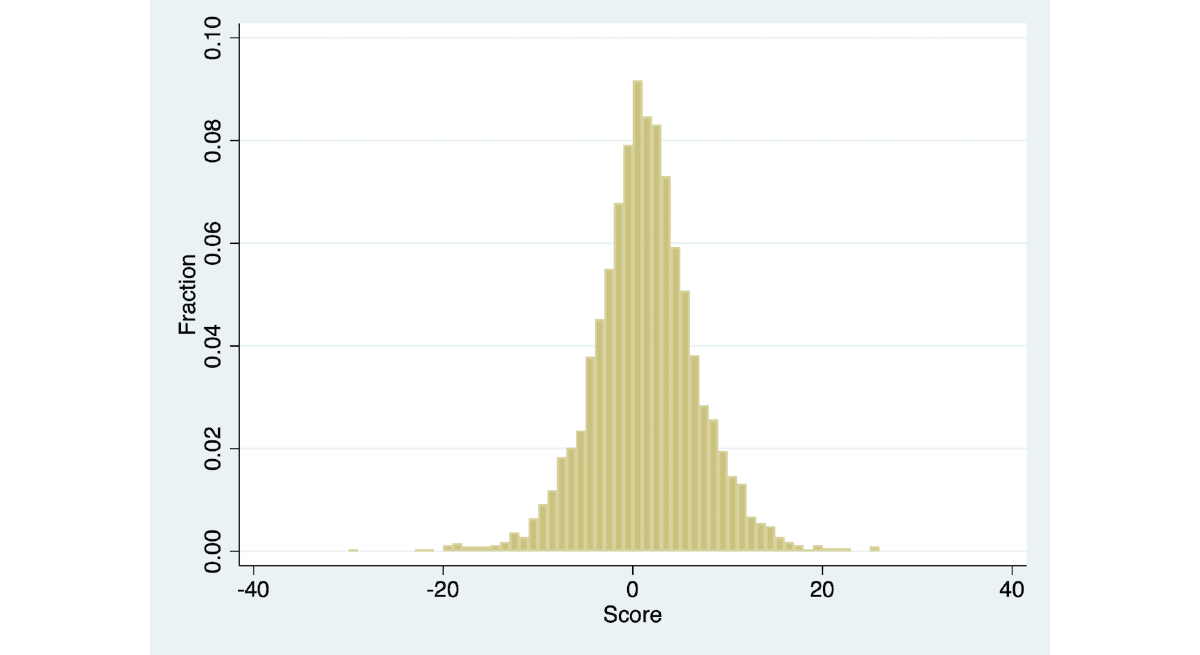
Distribution of changes in resilience at follow-up (full sample). N=3272; mean=.77; SD=5.5; median=1.

Figures 3 and 4 show the distribution of baseline resilience and changes in resilience, respectively, for the full sample of 3272 members. Baseline resilience was centered at 24 out of 40 points (mean 23.83, SD 6.47; median 24). The distribution is similar to a normal distribution; however, there is an excess mass toward the upper limit of the distribution and a relatively long left tail. We did not observe any evidence of scores being concentrated at any particular part of the distribution. Change in resilience was centered at 1 out of 40 points (mean 0.77, SD 5.50; median 1). The distribution was roughly normal, with a small number of outliers at either end of the distribution. All subsequent analyses were conducted including these outliers and excluding members below the fifth percentile for baseline resilience, below the fifth percentile for changes in resilience, and above the fifth percentile for changes in resilience. All the results were robust to excluding these outliers. For the sake of transparency, we presented the results inclusive of outliers.

Table 2 presents the number of members and statistics for members’ baseline resilience scores and score changes at follow-up for the overall sample and subgroups based on demographic characteristics and mental health outcomes at baseline.

**Table 2:** Resilience score characteristics by overall sample and subgroups group.

Characteristics		Participants, n (%)	Resilience score		Resilience change	
			Mean (SD)	*P* value	Mean (SD)	*P* value
All		3272 (100)	23.83 (6.47)	—^a^	0.77 (5.50)	—
**Gender**				.19		.14
	Female	1377 (42.08)	23.95 (6.49)		0.67 (5.49)	
	Male	569 (17.38)	24.39 (6.84)		1.07 (5.56)	
	Missing gender	1326 (40.52)	23.45 (6.26)	.005	0.74 (5.49)	.83
**Age (years)**				.01		.34
	18 to 24	176 (5.37)	22.84 (5.94)		0.27 (5.16)	
	25 to 34	844 (25.79)	23.76 (6.10)		0.54 (5.23)	
	35 to 44	547 (16.71)	24.54 (6.76)		0.94 (5.37)	
	45 to 64	461 (14.08)	24.29 (6.49)		0.89 (5.58)	
	≥65	37 (1.13)	25.03 (6.49)		0.14 (5.77)	
	Missing age	1207 (36.88)	23.48 (6.61)	.02	0.91 (5.75)	.28
**Region**				.54		.40
	West	1052 (32.15)	23.69 (6.06)		0.64 (5.22)	
	Midwest	290 (8.86)	23.93 (6.25)		0.80 (5.55)	
	South	994 (30.37)	23.94 (6.83)		1.03 (5.81)	
	Northeast	397 (12.13)	24.23 (6.46)		0.65 (5.33)	
	Missing region	539 (16.47)	23.53 (6.69)	.26	0.60 (5.56)	.45
**Baseline resilience**				<.001		<.001
	High resilience (CD-RISC-10^b^≥30)	598 (18.27)	33.15 (2.84)		–1.54 (5.11)	
	Low resilience (CD-RISC-10<30)	2674 (81.72)	21.74 (5.06)		1.28 (5.45)	
**Baseline depressive symptoms**				<.001		.31
	PHQ-9^c^ score≥10	1477 (45.14)	21.71 (6.38)		0.66 (5.78)	
	PHQ-9 score<10	1795 (54.85)	25.56 (6.01)		0.86 (5.26)	
**Baseline anxiety symptoms**				<.001		.55
	GAD-7^d^ score≥10	1327 (40.55)	21.70 (6.44)		0.70 (5.75)	
	GAD-7 score<10	1945 (59.44)	25.28 (6.07)		0.82 (5.33)	
**Engagement level**				<.001		<.001
	Self-guided	499 (15.25)	24.33 (6.66)		–0.11 (5.64)	
	Low engagement	989 (30.22)	23.87 (6.64)		0.52 (5.60)	
	Coaching only	544 (16.62)	24.61 (6.36)		1.26 (5.03)	
	Clinical only	670 (20.47)	23.45 (6.29)		1.06 (5.35)	
	Hybrid care	570 (17.42)	22.99 (6.18)		1.15 (5.70)	

^a^*P* values are reported only for testing differences in baseline resilience across categories of subgroups.

^b^CD-RISC-10: 10-item Connor-Davidson Resilience Scale.

^c^PHQ-9: 9-item Patient Health Questionnaire.

^d^GAD-7: 7-item Generalized Anxiety Disorder.

### Demographics

Demographic data were missing for a large portion of the sample because of irregular reporting by members’ employers or health plans. Of those without missing demographic data, most participants were female (1377/1946, 70.76%) and aged ≥35 years (1045/2065, 50.61%). Members were most likely to live in the West (1052/2733, 38.49%) and South (994/2733, 36.37%); however, all 4 census regions were represented in the baseline sample.

The baseline statistics and changes at follow-up are presented in Table 2. Columns 3 to 4 correspond to the baseline scores, and columns 5 to 6 correspond to the changes at follow-up. For each category (gender, age, etc), a *P* value is presented in the category’s first row to test whether the difference in mean changes in scores across the category was statistically significant. In the row for groups with missing data, the *P* value corresponds to a 2-tailed *t* test of the difference in mean baseline scores between those with and without missing data.

For categories based on gender and census region, neither the mean baseline resilience score nor the mean changes at follow-up were statistically different across groups (all *P*>.05). The mean baseline resilience score across age groups was significantly different, with older members having higher baseline resilience scores. Differences in the mean change in resilience across age groups were not statistically significant. However, the mean baseline scores for members with missing gender and age data were significantly different from those without missing data. This pattern did not hold for mean changes at follow-up (ie, members with missing demographic data did not have significantly different mean changes at follow-up than those without missing data).

### Mental Health Outcomes

The vast majority (2674/3272, 81.72%) of members reported low resilience at baseline (ie, CD-RISC-10 score<30). On the basis of the PHQ-9 and GAD-7 scores at baseline, 45.14% (1477/3272) of the members screened positive for clinical depression at baseline (PHQ-9 score≥10) and 40.55% (1327/3272) for clinical anxiety at baseline (GAD-7 score≥10). Consistent with prior work [10,11], the differences in mean baseline scores between the clinical and nonclinical groups were statistically significant. Specifically, the mean baseline scores for members who screened positive for either clinical depression (mean 21.71, SD 6.38) or anxiety (mean 21.7, SD 6.44) were significantly lower than for those who screened negative for depression (mean 25.56, SD 6.01) or anxiety (mean 25.28, SD 6.07). Members with subclinical depression and anxiety at baseline demonstrated an average resilience score improvement of 0.86 and 0.82 points, respectively; however, changes in resilience between those with clinical and nonclinical symptom scores was nonsignificant.

By construction, members with low baseline resilience scored below those with high resilience (mean 21.74 vs 33.15). On an average, members with low resilience at baseline demonstrated an increase of 1.28 points at follow-up. Conversely, members with high resilience at baseline evidenced decreasing scores (−1.54 points on average, SD 5.11) at follow-up. The difference in mean resilience score changes between these groups was significant (*P*<.001).

### Engagement Level

The most common engagement level was low engagement, with 30.22% (989/3272) of members meeting the criteria. There were 15.25% (499/3272) of members in the *self-guided* group, 16.62% (544/3272) of members in the *coaching only* group, 20.47% (670/3272) of members in the *clinical only* group, and 17.42% (570/3272) of members in the *hybrid care* group. Both baseline resilience and changes in resilience differed significantly across the groups based on engagement levels (both *P*<.001). Specifically, members in the *coaching only* group had the highest mean baseline resilience scores (mean 24.61, SD 6.36) and the largest mean change in resilience (mean 1.26, SD 5.03). The *hybrid care* group had the lowest mean baseline resilience (mean 22.99, SD 6.18), and the *self-guided* group had the lowest mean change in resilience (mean −0.11, SD 5.64).

Table 3 presents the results of the multivariate OLS regressions predicting changes in resilience at follow-up.

Gender predicted changes in resilience scores when controlling for all baseline characteristics; male participants (mean 1.07, SD 5.56) had significantly larger mean improvements in resilience scores than females (mean 0.67, SD 5.49; coefficient=0.58; *P*=.04). Although Table 2 shows that the mean change in scores for members with missing gender data was significantly different from those without missing data (not controlling for other variables, importantly, a member’s parent organization), Table 3 shows that these members are not associated with significantly different changes in scores. Similarly, age and census region did not predict changes in resilience scores when controlling for other variables.

Baseline resilience score was a strong predictor of changes in resilience scores at follow-up. Specifically, controlling for other variables, for each 1-point increase in baseline resilience score, the follow-up score decreased by 0.28 points, which was statistically significant at the 1% level. Baseline depression and anxiety were also strong predictors of changes at follow-up. Specifically, members without clinical depression or anxiety at baseline had mean resilience improvements of 1.44 points more than members with both clinical depression and anxiety (*P*<.001).

**Table 3:** Ordinary least squares regression of resilience change scores.

		β (95% CI)	*P* value
**Gender**			
	Female	Reference	Reference
	Male	0.58 (0.02 to 1.14)	.04
	Missing gender	–1.44 (–6.44 to 3.56)	.57
**Age (years)**			
	18 to 24	Reference	Reference
	25 to 34	0.10 (–0.74 to 0.95)	.81
	35 to 44	0.55 (–0.36 to 1.46)	.24
	45 to 64	0.59 (–0.36 to 1.53)	.22
	≥65	0.28 (–1.78 to 2.35)	.79
	Missing age	0.22 (–2.46 to 2.89)	.87
**Region**			
	West	Reference	Reference
	Midwest	0.08 (–0.91 to 1.07)	.88
	South	0.55 (–0.12 to 1.21)	.11
	Northeast	–0.30 (–1.12 to 0.52)	.47
	Missing Region	–0.30 (–1.53 to 0.94)	.64
Baseline resilience score		–0.28^b^ (–0.31 to –0.25)	<.001
**Baseline depressive and anxiety symptoms**			
	PHQ-9^a^ score≥10; GAD-7^b^ score≥10	Reference	Reference
	PHQ-9 score≥10; GAD-7 score<10	0.07 (–0.57 to 0.71)	.83
	PHQ-9 score<10; GAD-7 score ≥10	0.37 (–0.37 to 1.10)	.33
	PHQ-9 score<10; GAD-7 score<10	1.44 (0.96 to 1.93)	<.001
R-squared		0.1568234 (—^c^)	—
Adjusted R-squared		0.0972076 (—)	—
Observations		3272 (—)	—

^a^PHQ-9: 9-item Patient Health Questionnaire.

^b^GAD-7: 7-item Generalized Anxiety Disorder.

^c^Not available.

### Engagement Level and Moderator Analysis

**Figure 5:**
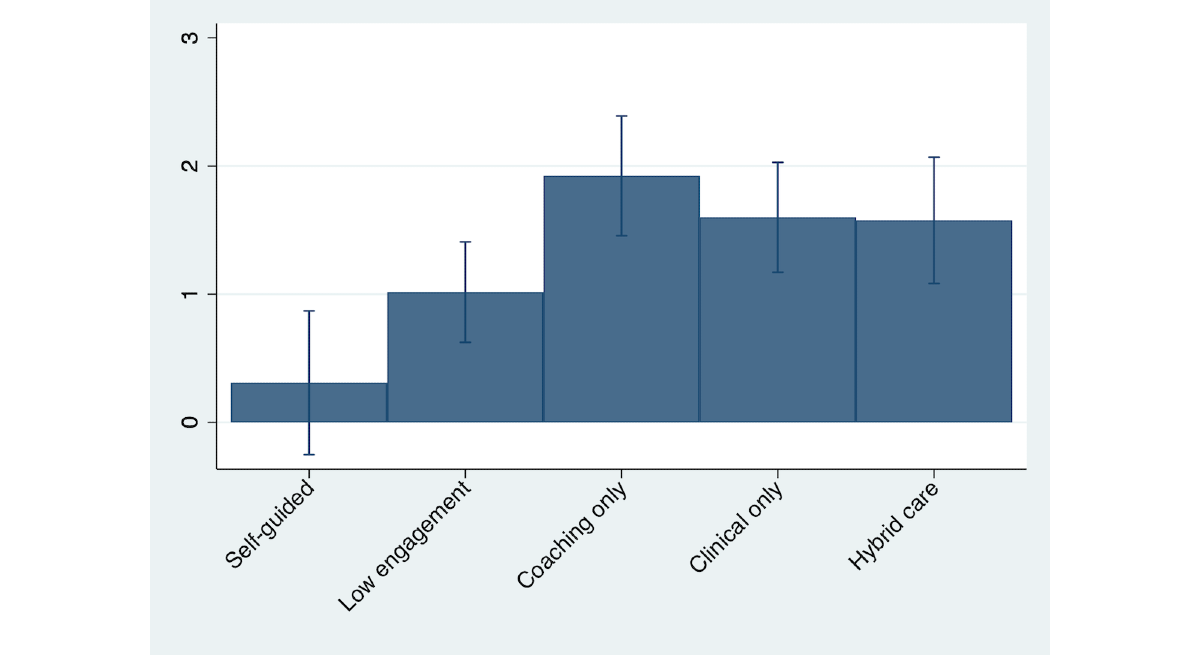
Change in resilience by engagement level (low resilience sample).

Figure 5 presents mean changes in resilience by the level of engagement received by a member between their baseline and follow-up scores. These results were restricted to members with low baseline resilience. Multimedia Appendix 1 presents similar results for the full sample. Members with self-guided engagement did not evidence significant improvements in mean resilience scores, based on a 5% significance level. Members with low engagement had significant improvements in resilience scores by approximately 1.0 points, whereas members with meaningful engagement (coaching only, clinical only, or hybrid care) had statistically significant improvements between 1.5 and 2.0 points between baseline and follow-up.

Table 4 reports the corresponding mean changes from a multivariate regression with the same set of independent variables included in the previous section. Table 4 includes members with low baseline resilience. Multimedia Appendix 2 Table S1 presents regression results for the full sample. Members with self-guided services had the smallest changes among all members. Members with low engagement saw changes in resilience scores that are 0.91 points larger than those who completed self-guided services (*P*=.01), whereas members engaged with coaching only, clinical only, or hybrid care services saw changes that are 1.82, 1.55, and 1.40 points larger than the self-guided group, respectively (all *P*<.001). Given the 95% CIs for the estimated coefficients on the indicators for low engagement, coaching only, clinical only, and hybrid care engagement levels, we cannot reject the null hypothesis at the 5% level that changes in resilience are the same across these levels of engagement.

**Figure 6:**
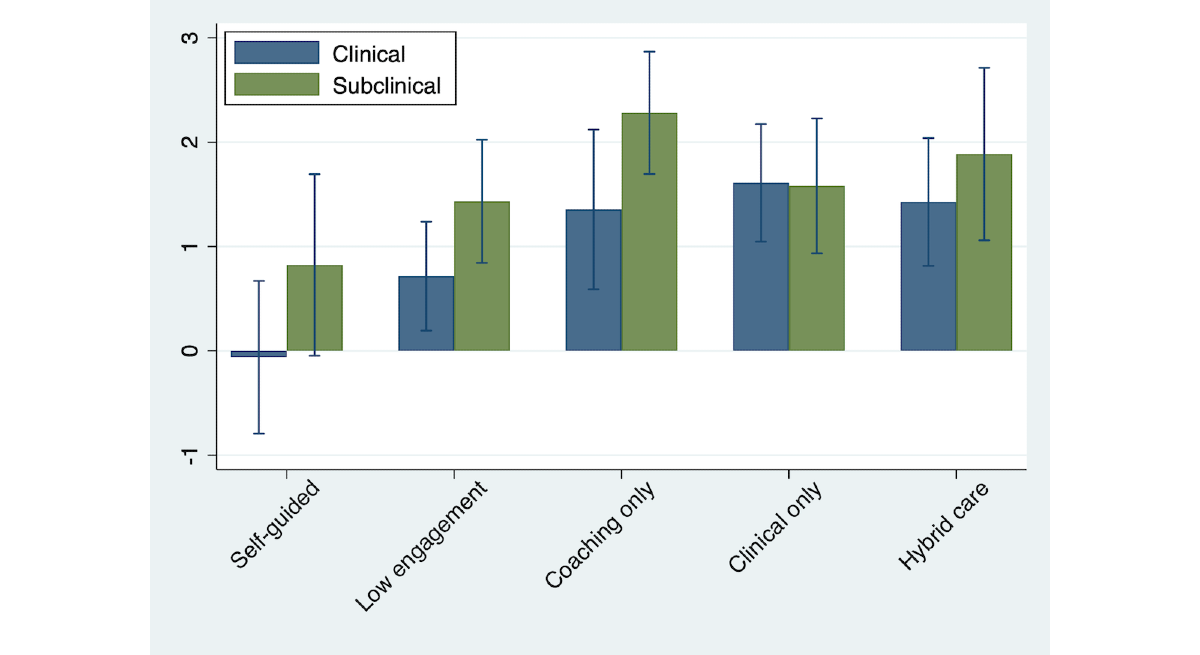
Change in resilience by engagement level and subclinical status (low resilience sample).

Figure 6 presents the same mean changes as Figure 5 but separately for subclinical members (ie, GAD-7 and PHQ-9 scores<10) and clinical members (ie, GAD-7 or PHQ-9 scores≥10). Figure 6 includes members with low baseline resilience. Multimedia Appendix 3 presents similar results for the full sample. Mean changes in resilience for clinical members were smaller than their subclinical counterparts across all engagement levels, except clinical only, where clinical and subclinical members saw similar improvements. On the basis of the 95% CIs around these means, the differences between clinical and subclinical members’ resilience score changes were not statistically different within each engagement level.

Table 5 presents the results from an interacted moderator model to formally test whether subclinical status at baseline is a moderator for the association between engagement level and changes in resilience. Table 5 includes members with low baseline resilience. Multimedia Appendix 2 Table S2 presents regression results for the full sample. Interacted terms refer to the interaction between the multinomial engagement level variable, which takes 1 of 5 values depending on a member’s engagement and an indicator for members who screen negative for both depression and anxiety at baseline. The coefficients of the indicators for engagement level were similar to the model without interactions, indicating that engagement level predicted changes in resilience for clinical members. The coefficient of the indicator for subclinical status interacted with the indicator for self-guided engagement was positive and statistically significant (coefficient=1.39; *P*=.02), indicating that subclinical status is associated with larger improvements in resilience for that engagement level. The coefficients of the regressors interacting engagement level with subclinical status were not significant, indicating that subclinical members’ improvements in resilience did not vary by engagement level. This indicates that subclinical members improved by roughly 1.4 points more than their clinical counterparts, regardless of their engagement level, thus rejecting the hypothesis that subclinical status is a moderator of the association between engagement level and changes in resilience.

**Table 4:** Ordinary least squares regression of changes in resilience scores (low resilience sample).

			β (95% CI)		*P* value
**Engagement level**					
	Self-guided	Reference		Reference	
	Low engagement	0.91 (0.20-1.63)		.01	
	Coaching only	1.82 (1.05-2.59)		<.001	
	Clinical only	1.55 (0.78-2.32)		<.001	
	Hybrid care	1.40 (0.61-2.19)		<.001	
Subclinical, both			1.30 (0.85-1.75)		<.001
R-squared			0.1357372 (—^a^)		—
Adjusted R-squared			0.0658413 (—)		—
Observations			2674 (—)		—

^a^Not available.

**Table 5:** Ordinary least squares regression of changes in resilience scores, interacted model (low resilience sample).

Engagement level		β (95% CI)	*P* value
**Self-guided**		Reference	Reference
	Interacted with subclinical	1.39 (0.21 to 2.57)	.02
**Low engagement**		0.83 (–0.11 to 1.77)	.08
	Interacted with subclinical	0.19 (–1.25 to 1.63)	.80
**Coaching only**		1.67 (0.55 to 2.79)	.003
	Interacted with subclinical	0.21 (–1.33 to 1.76)	.79
**Clinical only**		1.74 (0.76 to 2.71)	<.001
	Interacted with subclinical	−0.51 (–1.97 to 0.95)	.49
**Hybrid care**		1.53 (0.54 to 2.52)	.002
	Interacted with subclinical	−0.39 (–1.98 to 1.20)	.63
R-squared		0.1364253 (—^a^)	—
Adjusted R-squared		0.0650729 (—)	—
Observations		2674 (—)	—

^a^Not available.

## Discussion

### Principal Findings

The purpose of this study was to examine changes in resilience in members seeking on-demand mental health treatment as a function of both baseline symptoms of anxiety and depression, as well as engagement level. At baseline, most members (2674/3272, 81.72%) reported low resilience scores (ie, CD-RISC-10<30; mean 23.83, SD 6.47), which was well below the benchmarks of the US general population [29]. Overall, members experienced an average improvement in resilience of 0.77 points with a large variance around the mean (SD 5.50). According to the results by engagement level, members in the coaching only group had the largest changes in mean resilience between baseline and follow-up (mean 1.26, SD 5.03), followed by those in the hybrid care group (mean 1.15, SD 5.03). Across all levels of engagement, members who did not present with moderate to severe depression or anxiety at baseline saw larger improvements than their clinical counterparts (a difference of 1.3 points; *P*<.001, when controlling for covariates). Given that the difference between clinical and subclinical members’ improvements did not vary significantly across levels of engagement, we conclude that subclinical status is not a moderator for the association of levels of engagement with changes in resilience. That is, although subclinical members improved more than clinical members, the gap between these groups was stable across all levels of engagement.

In subgroup analyses, members with low resilience at baseline, on average, demonstrated a 1.28-point improvement in resilience scores at follow-up. Resilience has been conceptualized as a dynamic process, and engagement with coaching or teletherapy may contribute to improvements in resilience, even in the absence of it being the focus of services [4,20,30]. Conversely, members with high resilience scores at baseline demonstrated a decrease in the CD-RISC-10 at follow-up (mean −1.54, SD 5.11). These findings could be a function of several factors, including regression to the mean or recalibration or self-discovery by members as part of treatment. Higher scores may leave less room for improvement. Prior research has shown that there is a ceiling effect in being able to detect improvement in individuals who self-report high resilience at baseline [31]. In addition, these findings could be the result of selection or attrition bias. Specifically, those with high resilience who continue to use treatment services may have a higher need for exogenous factors that impact resilience. On the basis of the results of the regression analyses, these findings could also be driven by members who reported high resilience (ie, CD-RISC-10 >30) but also presented with clinical depression or anxiety.

The construct of resilience can vary as a function of time, context, gender, and age [7]. However, our study did not find any statistically significant differences across demographic groups overall (ie, gender and location), with the exception of baseline resilience by age group. Specifically, those in the youngest age group demonstrated significantly lower baseline resilience (mean 22.84, SD 5.94) compared with other age groups. Resilience has been found to be greater in older adults, particularly those with emotional regulation ability and problem-solving ability [32]. When controlling for other baseline variables, our regression analysis found that male participants had a significantly larger mean change in resilience scores than female participants, suggesting that one must consider the socioecological context and treatment process when examining changes in resilience, including gender. Hirani et al [33] suggested that women typically score lower on measures of resilience because existing conceptualizations of resilience are not reflective of how gender roles, social expectations, perceptions, and environmental factors, among others, interact to differentially impact experiences for men and women and, in turn, their response to adversity.

We found that members with symptom scores within the clinical range (ie, PHQ-9 or GAD-7 score≥10) demonstrated significantly lower baseline resilience scores. When controlling for other baseline characteristics, members without clinical depression or anxiety at baseline had improvements in resilience of 1.4 points more than members with both clinical depression and anxiety. Given the negative association between resilience and clinical symptom severity [10,11], ongoing symptomatology may negatively affect changes in resilience. Moreover, members with clinical symptomatology may focus on symptom management before transitioning to a focus on positive psychological outcomes such as resilience. One may expect that improvements in mental health symptom scores would parallel changes in resilience over the course of treatment. In addition, resilience-focused interventions may serve as a preventive approach to reduce the exacerbation of mental health symptoms that would meet the clinical threshold.

Regarding the association between care engagement level and both baseline and changes in resilience, there were significant differences in the mean baseline resilience and changes in resilience between those with and without meaningful engagement (ie, coaching only, clinical only, or hybrid care vs self-guided or minimal engagement). Specifically, members with self-guided engagement did not show any improvement in resilience, followed by those with minimal engagement having a 0.5-point improvement on average. Those with meaningful engagement (ie, coaching only, clinical only, or hybrid care) demonstrated improvements in resilience between 1.06 and 1.26 points. Within the Ginger context, these findings support the hypothesis that engagement with a human care provider (which is true for members in the coaching only, clinical only, and hybrid care groups) can lead to larger improvements in resilience than engagement with self-guided content alone. This is perhaps not surprising, given the more intensive nature of care delivered by a human provider. Behavioral health coaches, therapists, and psychiatrists tailor their care to the specific needs of a member, whereas it is incumbent on a member to find and engage with content that is applicable to their needs. This does not rule out that resilience-focused content could have an impact on resilience; however, given the wide variety of content available on the Ginger platform, this study was not designed to test whether resilience-specific content was associated with greater or lesser improvements in resilience than the care provided by a trained human provider. These findings are not surprising, as previous research has demonstrated that individuals who had greater engagement in a web-based resilience training program achieved the greatest improvements [10].

Controlling for baseline characteristics, members with subclinical status at baseline had larger improvements in resilience across all engagement levels; however, our moderation analysis found that the association between care engagement and changes in resilience did not significantly differ by baseline subclinical status.

### Limitations

From a research perspective, there is much ambiguity in approaching the concept of resilience and no current consensus on the operational definition of resilience [12]. Moreover, the construct is sometimes assessed as a dynamic process and at other times as a personal trait or an outcome [4,13]. For example, some conceptualize resilience as the ability to *bounce back* from adversity, conflict, and failure [30]. Future research is needed to develop greater conceptual clarity around resilience, particularly as it relates to coaching interventions for depression and anxiety. This definition and conceptualization of resilience should fit the behavioral health coaching context [19]. There are many validated tools to assess resilience, in addition to the one used in this study (eg, the Resilience Scale for Adults, Psychological Capital Questionnaire, and Cognitive Hardiness Scale). Each of these tools reflects differing conceptualizations of resilience; thus, changes in resilience scores over time could capture different processes, with some being more static than others [19].

The construct of resilience can vary as a function of time, context, gender, age, and cultural origin [7]. A large percentage of the participants did not have age (1207/3272, 36.88%) or gender (1326/3272, 40.52%) reported and there was limited access to other demographic information of the study participants. Thus, we were unable to stratify analyses by key demographic factors or examine factors that could affect resiliency and treatment response (eg, marital status, family composition, significant life events, previous mental health treatment, sources of social support, and educational level). These analyses could provide additional insights into those who may best benefit from virtual care and those for whom additional support may be needed. In addition, because there was no comparison group in this retrospective observational study, we were unable to draw any causal inferences regarding the impact of the Ginger on-demand mental health platform.

Another limitation of this study is that baseline surveys for resilience, depression, and anxiety symptoms were conducted at different times. This could have led to measurement errors, as both types of outcomes could evolve before the other is measured. The lag between collecting CD-RISC-10 and PHQ-9 and GAD-7 (the latter 2 were collected at the same time) was intentional in an effort to avoid survey fatigue. Therefore, the results of this study should be interpreted with this lag in mind.

### Conclusions

This study examined changes in resilience over time among members of an on-demand virtual mental health system. Overall, members with low baseline resilience and subclinical symptoms of anxiety and depression demonstrated the largest improvement in resilience over time. Even for interventions that did not focus on resilience, members could demonstrate improvements in resilience over the course of treatment with virtual-based treatment for anxiety and depression. Future studies should examine symptom scores of anxiety and depression over time in relation to resilience. In addition, the inclusion of measures such as perceived social support might provide additional insight into treatment, given its association with both mental health and resilience [4,34].

## Appendix

Multimedia Appendix 1 Change in resilience by engagement level (full sample).

Multimedia Appendix 2 Additional results using the full sample.

Multimedia Appendix 3 Change in resilience by engagement level and subclinical status (full sample).

## Abbreviations

CD-RISC-10: 10-item Connor-Davidson Resilience Scale
GAD-7: 7-item Generalized Anxiety Disorder
OLS: ordinary least squares
PHQ-9: 9-item Patient Health Questionnaire

## References

[1] Windle G (2011). What is resilience? A review and concept analysis. Rev Clin Gerontol.

[2] Earvolino-Ramirez M (2007). Resilience: a concept analysis. Nurs Forum.

[3] Wald J, Taylor S, Asmundson GJ, Jang K, Stapleton JA (2006). Literature review of concepts: psychological resiliency. Defence R&D Canada – Toronto.

[4] Herrman H, Stewart DE, Diaz-Granados N, Berger EL, Jackson B, Yuen T (2011). What is resilience?. Can J Psychiatry.

[5] Feldman R (2020). What is resilience: an affiliative neuroscience approach. World Psychiatry.

[6] Ungar M, Theron L (2020). Resilience and mental health: how multisystemic processes contribute to positive outcomes. Lancet Psychiatry.

[7] Hu T, Zhang D, Wang J (2015). A meta-analysis of the trait resilience and mental health. Pers Individ Dif.

[8] Zadok-Gurman T, Jakobovich R, Dvash E, Zafrani K, Rolnik B, Ganz AB, Lev-Ari S (2021). Effect of Inquiry-Based Stress Reduction (IBSR) intervention on well-being, resilience and burnout of teachers during the COVID-19 pandemic. Int J Environ Res Public Health.

[9] Campbell-Sills L, Cohan SL, Stein MB (2006). Relationship of resilience to personality, coping, and psychiatric symptoms in young adults. Behav Res Ther.

[10] Riehm KE, Brenneke SG, Adams LB, Gilan D, Lieb K, Kunzler AM, Smail EJ, Holingue C, Stuart EA, Kalb LG, Thrul J (2021). Association between psychological resilience and changes in mental distress during the COVID-19 pandemic. J Affect Disord.

[11] Kermott CA, Johnson RE, Sood R, Jenkins SM, Sood A (2019). Is higher resilience predictive of lower stress and better mental health among corporate executives?. PLoS One.

[12] Liu JJ, Ein N, Gervasio J, Battaion M, Reed M, Vickers K (2020). Comprehensive meta-analysis of resilience interventions. Clin Psychol Rev.

[13] Smith B, Shatté A, Perlman A, Siers M, Lynch WD (2018). Improvements in resilience, stress, and somatic symptoms following online resilience training: a dose-response effect. J Occup Environ Med.

[14] Pearman A, Hughes ML, Smith EL, Neupert SD (2021). Age differences in risk and resilience factors in COVID-19-related stress. J Gerontol B Psychol Sci Soc Sci.

[15] Schuster C, Weitzman L, Sass Mikkelsen K, Meyer-Sahling J, Bersch K, Fukuyama F, Paskov P, Rogger D, Mistree D, Kay K (2020). Responding to COVID-19 through surveys of public servants. Public Adm Rev.

[16] Killgore WD, Cloonan SA, Taylor EC, Dailey NS (2020). Loneliness: a signature mental health concern in the era of COVID-19. Psychiatry Res.

[17] Ran L, Wang W, Ai M, Kong Y, Chen J, Kuang L (2020). Psychological resilience, depression, anxiety, and somatization symptoms in response to COVID-19: a study of the general population in China at the peak of its epidemic. Soc Sci Med.

[18] Ju G, Lee J, Ahn MH, Lee J, Kim EJ, Suh S, Chung S (2021). Effects of depression and resilience of public workers on work-related stress and anxiety in response to the COVID-19 pandemic. J Korean Med Sci.

[19] Smith CL, Bachkirova T, Spence G, Drake D (2017). Coaching for resilience and well-being. The SAGE Handbook of Coaching.

[20] Grant AM, Curtayne L, Burton G (2009). Executive coaching enhances goal attainment, resilience and workplace well-being: a randomised controlled study. J Posit Psychol.

[21] Lee JA, Heberlein E, Pyle E, Caughlan T, Rahaman D, Sabin M, Kaar JL (2021). Evaluation of a resiliency focused health coaching intervention for middle school students: building resilience for healthy kids program. Am J Health Promot.

[22] Kunkle S, Yip M, Ξ W, Hunt J (2020). Evaluation of an on-demand mental health system for depression symptoms: retrospective observational study. J Med Internet Res.

[23] Kunkle S, Yip M, Hunt J, Ξ W, Udall D, Arean P, Nierenberg A, Naslund JA (2021). Association between care utilization and anxiety outcomes in an on-demand mental health system: retrospective observational study. JMIR Form Res.

[24] Campbell-Sills L, Forde DR, Stein MB (2009). Demographic and childhood environmental predictors of resilience in a community sample. J Psychiatr Res.

[25] Vaishnavi S, Connor K, Davidson JR (2007). An abbreviated version of the Connor-Davidson Resilience Scale (CD-RISC), the CD-RISC2: psychometric properties and applications in psychopharmacological trials. Psychiatry Res.

[26] Kroenke K, Spitzer RL, Williams JB (2001). The PHQ-9: validity of a brief depression severity measure. J Gen Intern Med.

[27] Spitzer RL, Kroenke K, Williams JB, Löwe B (2006). A brief measure for assessing generalized anxiety disorder: the GAD-7. Arch Intern Med.

[28] Plummer F, Manea L, Trepel D, McMillan D (2016). Screening for anxiety disorders with the GAD-7 and GAD-2: a systematic review and diagnostic metaanalysis. Gen Hosp Psychiatry.

[29] Wingo AP, Briscione M, Norrholm SD, Jovanovic T, McCullough SA, Skelton K, Bradley B (2017). Psychological resilience is associated with more intact social functioning in veterans with post-traumatic stress disorder and depression. Psychiatry Res.

[30] Tugade MM, Fredrickson BL (2004). Resilient individuals use positive emotions to bounce back from negative emotional experiences. J Pers Soc Psychol.

[31] Cameron D, Dromerick LJ, Ahn J, Dromerick AW (2019). Executive/life coaching for first year medical students: a prospective study. BMC Med Educ.

[32] Gooding PA, Hurst A, Johnson J, Tarrier N (2012). Psychological resilience in young and older adults. Int J Geriatr Psychiatry.

[33] Hirani S, Lasiuk G, Hegadoren K (2016). The intersection of gender and resilience. J Psychiatr Ment Health Nurs.

[34] Li F, Luo S, Mu W, Li Y, Ye L, Zheng X, Xu B, Ding Y, Ling P, Zhou M, Chen X (2021). Effects of sources of social support and resilience on the mental health of different age groups during the COVID-19 pandemic. BMC Psychiatry.

