# Supplementary figures and images for: Changes in Resilience Following Engagement With a Virtual Mental Health System: Real-world Observational Study
Source: JMIR Form Res. 2022 Jul 29;6(7):e37169. doi: 10.2196/37169 (PMC9377433; doi:10.2196/37169)

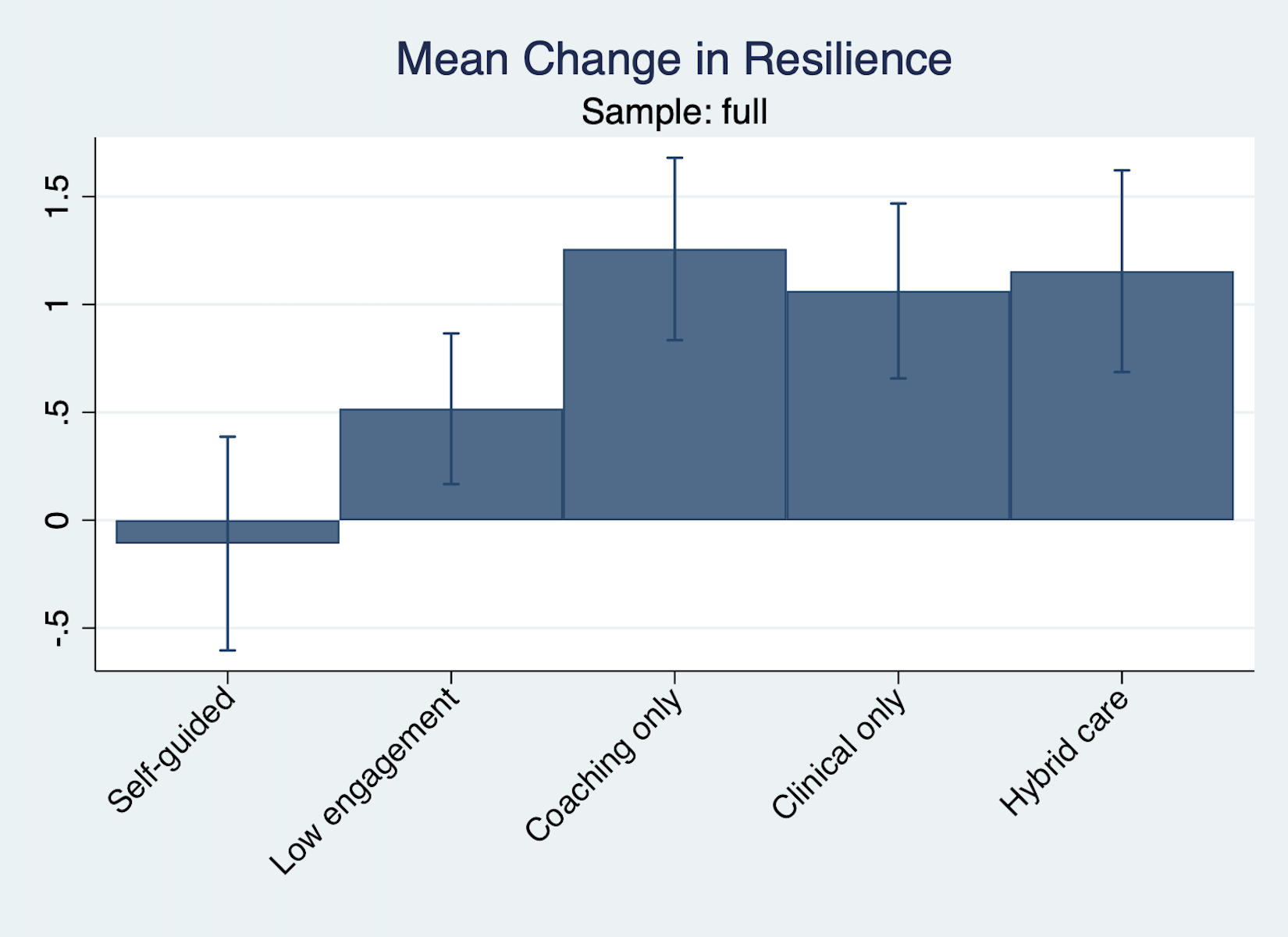

Supplement: Multimedia Appendix 1 [file formative_v6i7e37169_app1.png]

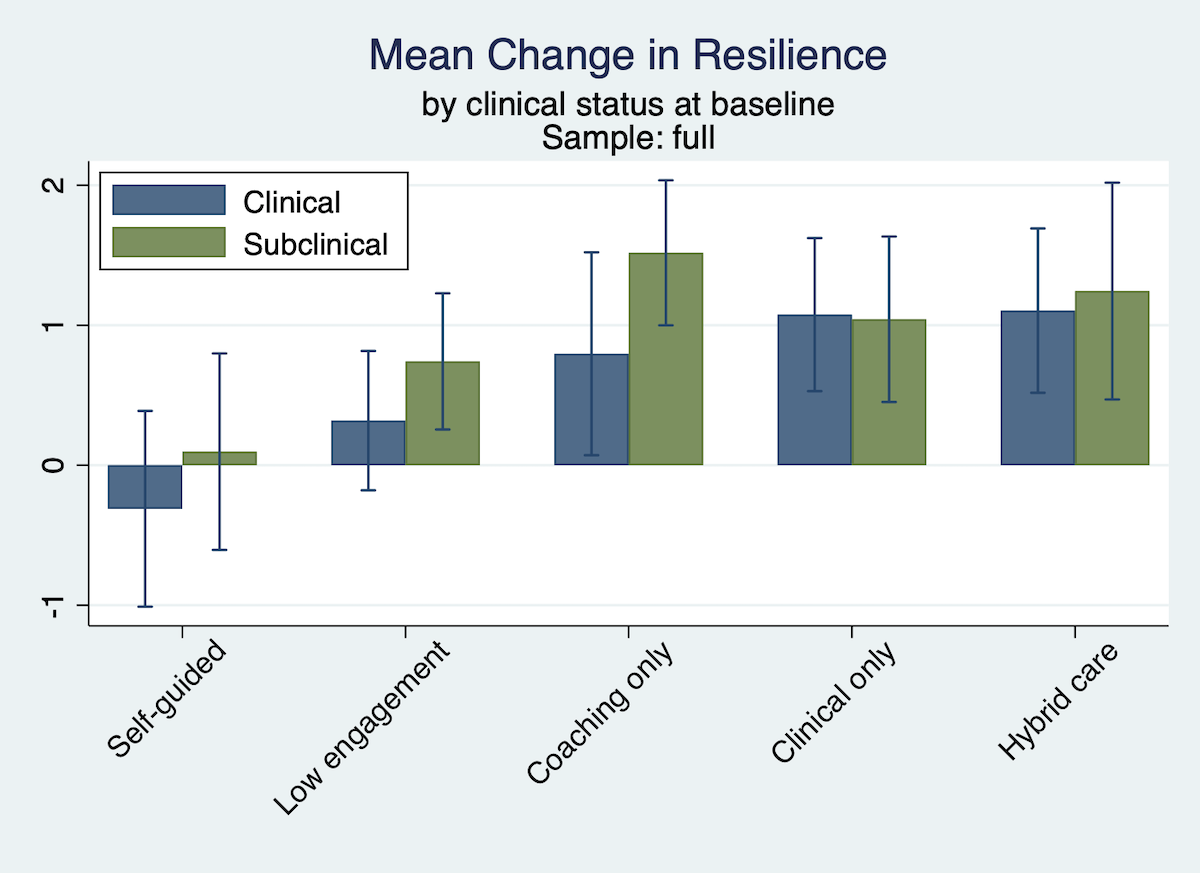

Supplement: Multimedia Appendix 3 [file formative_v6i7e37169_app3.png]
