# Supplementary material for: Changes in Resilience Following Engagement With a Virtual Mental Health System: Real-world Observational Study
Source: JMIR Form Res. 2022 Jul 29;6(7):e37169. doi: 10.2196/37169 (PMC9377433; doi:10.2196/37169)
Supplement: Multimedia Appendix 2 [file formative_v6i7e37169_app2.docx]

# **Appendix 2**

The appendix includes additional results using the full sample.

**Table S1.** Ordinary least squares regression changes in resilience scores (full sample)

|  | $\beta$ (95% CI) | P value |
| --- | --- | --- |
| **Engagement level** |  |  |
| Self-guided | Reference | Reference |
| Low engagement | 0.79 (0.18 to 1.14) | .012 |
| Coaching only | 1.56 (0.89 to 2.23) | <.001 |
| Clinical only | 1.32 (0.64 to 1.99) | <.001 |
| Hybrid | 1.33 (0.63 to 2.03) | <.001 |
|  | | |
| Subclinical, both | 1.38 (0.97 to 1.78) | <.001 |
|  | | |
| R-squared | 0.1601258 |  |
| Adjusted R-squared | 0.1016258 |  |
| Observations | 3272 |  |

**Table S2.** Ordinary least squares regression changes in resilience scores, interacted model (full sample)

|  | Coefficient $\beta$ | P value |
| --- | --- | --- |
| **Engagement level** |  |  |
| Self-guided | Reference | Reference |
| Interacted with subclinical | 1.58 (0.56 to 2.61) | .002 |
| Low engagement | 0.76 (0.12 to 1.63) | .09 |
| Interacted with subclinical | 0.08 (-1.15 to 1.31) | .90 |
| Coaching only | 1.59 (0.56 to 2.62) | .003 |
| Interacted with subclinical | -0.09 (-1.44 to 1.26) | .90 |
| Clinical only | 1.56 (0.66 to 2.46) | .001 |
| Interacted with subclinical | -0.58 (-1.86 to 0.70) | .38 |
| Hybrid | 1.55 (0.63 to 2.47) | .001 |
| Interacted with subclinical | -0.55 (-1.97 to 0.87) | .45 |
|  | | |
| R-squared | 0.16074 |  |
| Adjusted R-squared | .10111 |  |
| Observations | 3272 |  |

**Figure S1.** *Change in resilience by engagement level (full sample)*

*
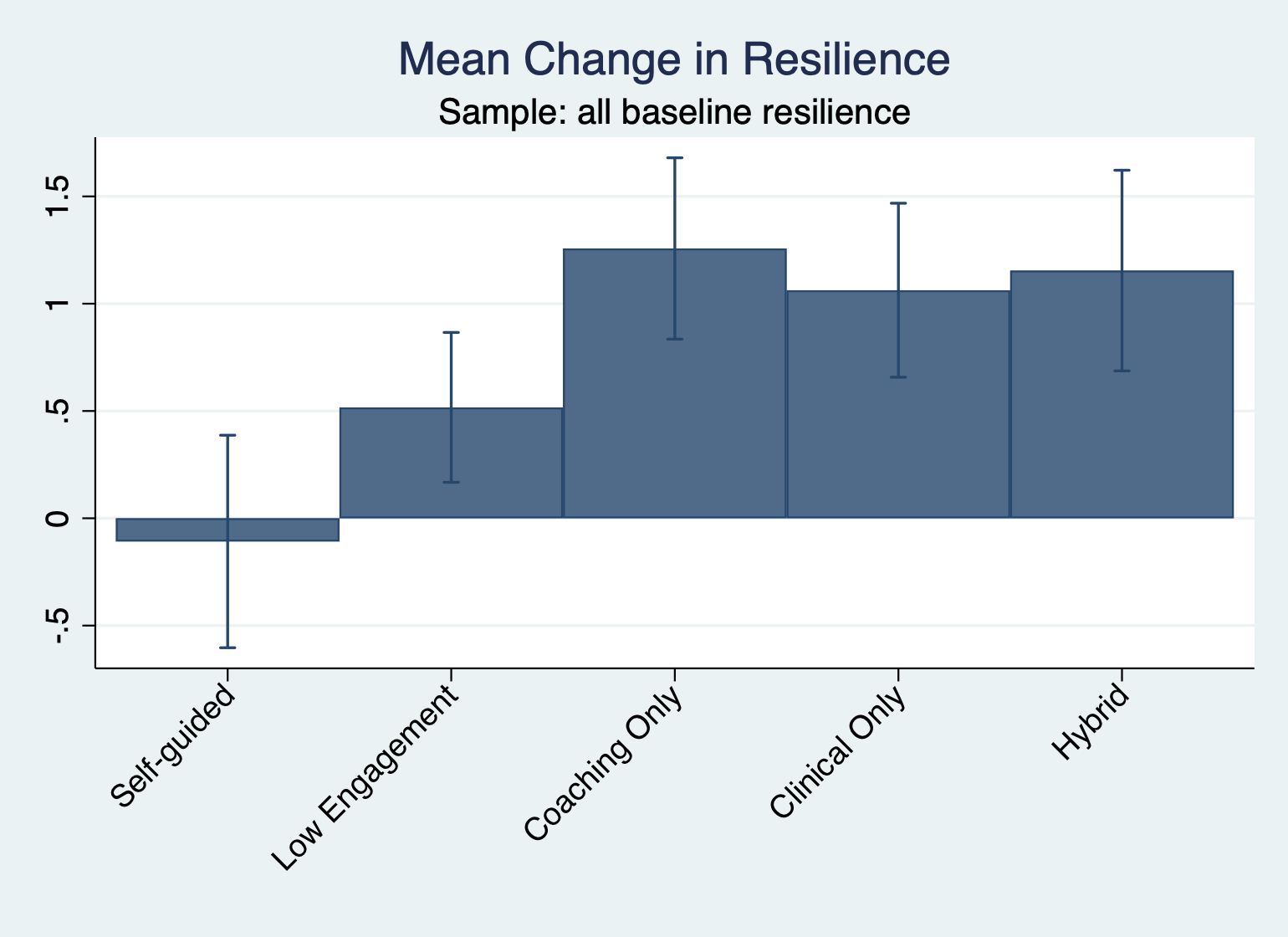
*

**Figure S2.** *Change in resilience by engagement level and subclinical status (full sample)*

*
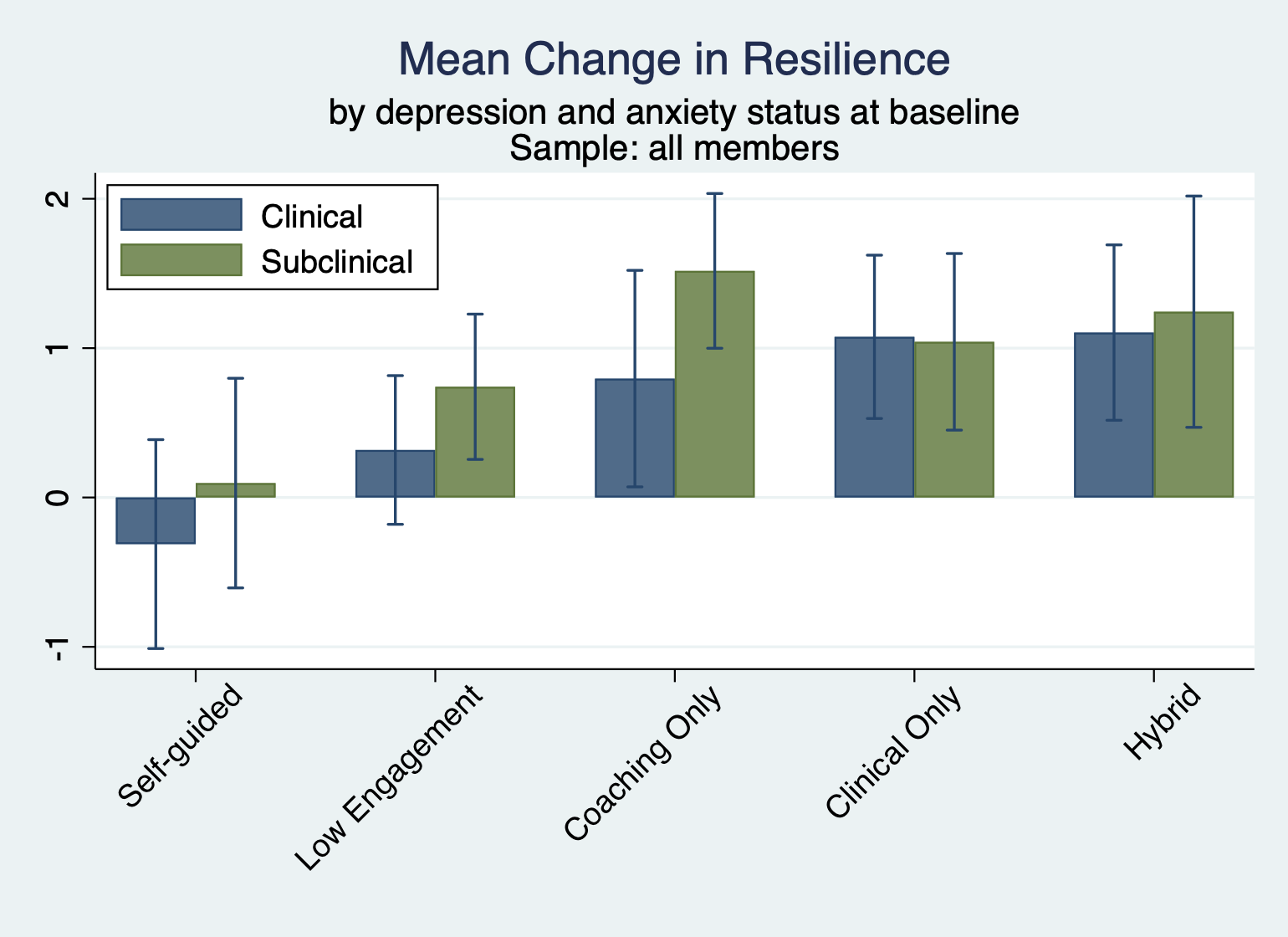
*
